# Development of Dressing-Type Emulsion with Hydrocolloids from Butternut Squash Seed: Effect of Additives on Emulsion Stability

**DOI:** 10.3390/gels8040209

**Published:** 2022-03-30

**Authors:** Somaris E. Quintana, Edilbert Torregroza-Fuentes, Luis A. García Zapateiro

**Affiliations:** 1 Research Group of Complex Fluid Engineering and Food Rheology, University of Cartagena, Cartagena 130015, Colombia; squintanam@unicartagena.edu.co; 2 Research Group Science Technology and Society—CTS, University of Cartagena, Cartagena 130015, Colombia; etorregrozaf@unicartagena.edu.co

**Keywords:** butternut squash seed, hydrocolloids, dressing-type emulsion, rheological properties

## Abstract

Background: Natural ingredients have been employed to develop food products. Methods: Hydrocolloids from butternut squash seeds (HBSSs) were extracted with water at pH 3, 7, and 10 and characterized bromatologically and rheologically; then these HBSSs were used to stabilize the dressing-type emulsion by evaluating its physicochemical, rheological, and microstructural properties. Results: Hydrocolloids presented higher protein (from 20.43 to 39.39%) and carbohydrate (from 50.05 to 52.68%) content and rheological properties with a predominant elastic modulus. HBSSs extracted at pH 10 were used for the development of the dressing-type emulsion. The samples were stable during the storage period (15 days), with a good microstructural organization showing non-Newtonian fluid properties with shear-thinning behavior when the pseudoplasticity and the oil droplet size decreased with the addition of HBSS. Conclusions: Hydrocolloid constituents were detected surrounding the droplets of the emulsions, intensifying the effects of inner droplet interaction due to depletion events and a strong influence on the structure and physical stability. The hydrocolloids used to stabilize the dressing-type emulsions are additively promising in microstructured food design.

## 1. Introduction

Several food products, such as dressing-type emulsions and mayonnaise, are oil in water (o/w) emulsions, wherein oil droplets are dispersed in an aqueous phase, exhibiting a creamy texture characteristic. The emulsions are thermodynamically unstable systems and could show phase separation through different physicochemical processes, such as gravitational separation, coalescence, flocculation, and Ostwald ripening [1]. These are two types of additives that are often used to avoid phase separation. The use of additives helps to increase the stability of aging. The emulsion needs an emulsifier to adsorb at the oil–water interface and create a film around the oil droplets that ensures their physicochemical stability [2], as well as stabilizers, increasing the viscosity of the continuous medium. Both types of molecules may be adsorbed at fluid interfaces, reducing the interfacial tension, thus facilitating the formation of emulsions by providing stability to freshly formed droplets [3].

Food emulsions can exhibit a variety of rheological characteristics, depending mainly on the level of emulsifiers, stabilizers, and volume fraction of the oil phase [4]; then, when the oil content is reduced below 60% to 65%, the emulsions become unstable, and the addition of a hydrocolloid is required to prevent undesirable separation of serum and oil during storage [5]. There are numerous types of emulsifiers used in the food industry, such as natural and synthetic emulsifiers [6], where the latter ones are the most commonly used due to their high effectiveness [1,7]. In the case of dressing-type emulsions, the nonionic surfactant tween family [8] and egg yolk [9,10] are added to the water phase of emulsions to stabilize them, preventing the coalescence of the droplets from the dispersed phase. Furthermore, a mixture of protein or hydrocolloid emulsifiers has been used in various studies; that is, Gavihian et al. [11] prepared a salad dressing emulsion by ultrasound homogenization with acacia gum and soybean lecithin, Diftis et al. [12] stabilized an emulsion with a protein isolate–dextran mixture, Ma et al. [13] with egg yolk-xanthan gum, Plati et al. [14] with egg yolk- Arabic gum-carboxymethyl cellulose-maltodextrin, and Martínez et al. [15] with egg yolk with pea protein, sucrose laurate, or Tween 20.

Food consumers’ demand for stabilized emulsions with natural emulsifiers and the industrial need for employing highly efficient emulsification techniques highlights the importance of investigating the effectiveness of natural emulsifiers and alternative emulsification techniques. However, the food industry has a growing trend to replace synthetic emulsifiers with natural components due to consumer concerns about health awareness [16], increasing interest in looking for new sources of natural ingredients.

Hydrocolloids are natural compounds, such as proteins and polysaccharides, with some hydrophilic groups to act as gelling agents, thickeners, coating, suspension dispersions, foams, and stabilizers for emulsions [17]. Due to their beneficial functional characteristics, hydrocolloids are used as a new food additive, gradually attracting attention in the food industry [18]. They are usually added at relatively low concentrations due to their high molecular weight and technological functionality [19].

Natural hydrocolloids are used in food emulsion products to stabilize or improve their rheological properties [19,20,21,22], such as pectin extracted from beet and okra [23,24] and water-soluble yellow mustard mucilage [25], as well as hydrocolloids from squash peel [26]. The physicochemical and functional properties of hydrocolloids are influenced by chemical composition, molecular structure, extraction, and subsequent processing [27,28].

Butternut squash is an unconventional source of dietary fiber, phenolic compounds, minerals, vitamins, proteins, and carbohydrates [28,29]. Squash seeds, generally considered agro-industrial waste [30], are an extraordinarily rich source of fiber (31.48% crude fiber) [31], proteins, essential fatty acids, sterols, arginine, vitamins, and trace elements [32]. In addition, they are composed of mannose, glucose, and galactose [33]. In this context, the research presented in this paper focuses on (1) obtaining and characterizing hydrocolloids from butternut squash seeds and (2) evaluating their application on the stabilization of dressing-type emulsions.

## 2. Results and Discussion

### 2.1. Hydrocolloids from Butternut Squash Seed

Different hydrocolloids were obtained from butternut squash seeds (HBSSs). The extraction yield (Ys %) value, proximal composition, and rheological properties are summarized in Table 1. In general, the extraction yield was significantly (*p* < 0.05) influenced by the pH of solubilization. The current study showed a wide range of extraction yields, with 4.68 ± 0.12, 21.17 ± 0.32, and 31.96 ± 0.27% for the extractions at pH 3, 7, and 10, respectively. This range was greater than the extraction yield reported for flaxseed gum (7.9%) [34], malva nut gum (20%) [35], and yanang gum (4.54%) [36]. The wide difference between extraction yields (Ys %) increased with the pH of solubilization, which presented the highest value at pH 10 (31.96 ± 0.27%), which showed that most polysaccharides are physically trapped in the cell wall matrix or covalently linked to other components, which can only be effectively released under alkaline conditions. Similar results were obtained by Karazhiyan et al. [37] and Somboonpanyakul et al. [35] when alkaline conditions increased Ys % by hydrolyzing insoluble constituents into soluble ones, which increased extraction yield.

All extraction was performed with an employed 1:10 seed: water ratio, ensuring that the presence of excessive water for aqueous extraction led to a greater binding of the water-soluble components present in the seed endosperm. In addition, the higher temperature employed (80 °C) is related to an increased solubility at higher temperatures [38,39] after a long hydration time [40,41], thereby resulting in higher extraction efficiency. In addition, extraction at high temperature results in the faster and easier mass transfer of water-soluble polysaccharides from the cell wall into the extract. Extraction at an elevated temperature may result in a broad range of constituents with different molecular mass [38].

The proximal composition (moisture, ash, protein, fat, and carbohydrates) shows that HBSS has the highest values of carbohydrates and protein. Hydrocolloids present similar carbohydrate values of carbohydrates (*p* > 0.05) between 50.05 and 52.60%; then extraction at pH 10 yielded the highest protein content (39.30 ± 0.21%) and lowest for pH 3 and 7 (29.43 ± 0.17 and 36.70 ± 0.38%, respectively). This indicates that more protein was extracted under alkali conditions. This protein was co-precipitated with the polysaccharide when alcohol was added; these results are according to Vinayashree and Vasu [42], establishing that the highest fraction of *Cucurbita* spp. protein could be obtained under alkali conditions due to solubilization.

To evaluate the rheological properties of hydrocolloids, an oscillatory analysis was performed. Dynamic shear analysis can express more about the structural behavior and the time, frequency, or temperature dependence. This information is required to predict the behavior of materials through processing and post-processing manipulations (i.e., mixing, pumping, emulsification, and shelf-life stability) [43].

**Figure 1:**
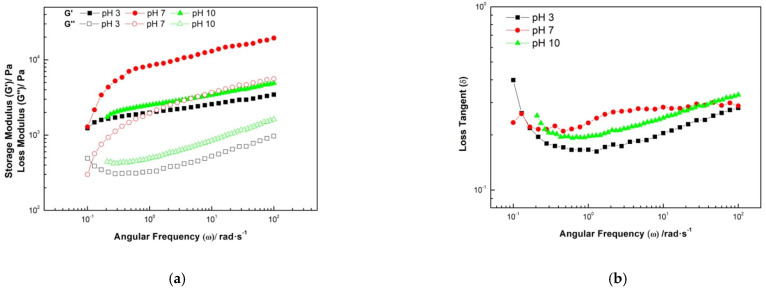
Dynamic rheology properties of hydrocolloids from butternut squash seeds at pH 3, 7, and 10. Frequency sweep (**a**) and loss tangent (**b**).

Comparison of the storage ($G^{\prime }$) and loss ($G^{\prime \prime }$) moduli values as a function of frequency (Figure 1a) is highly informative about the structural behavior of hydrocolloids and the strength of the network connections in solution matrices to predict the behavior of compounds within process operations or shelf life [43]. A frequency sweep (Figure 1a) showed the storage modulus ($G^{\prime }$) was higher than the loss modulus ($G^{\prime \prime }$), indicating that the elastic component was predominate. This type of mechanical spectra corresponds to a solution of entangled macromolecules, since gels would show values of $G^{\prime }$ higher than $G^{\prime \prime }$ throughout the frequency range [44]. Similar results were obtained for hydrocolloids from butternut peel by Quintana et al. [26], related to a strong association of cross-linked molecules within the diluted gum [45].

Therefore, some elastic behavior is expected from the extracted hydrocolloids, which is described by a relatively strong association of the cross-linked molecules within the diluted hydrocolloid solution network [45]. Then, the samples did not present significant differences in the loss tangent (Tan (δ)), with values closer to 0, presenting properties of solid-like behavior.

### 2.2. Dressing-Type Emulsion

#### 2.2.1. Physicochemical Properties

Considering hydrocolloids’ carbohydrate and protein content, the sample extracted at pH 10 was selected to prepare a dressing-type emulsion. Four samples were developed, evaluating the percentage of HBSS as shown in Table 2.

The samples were stable during the storage period (15 days), with a good microstructural organization. The results of the chemical composition analysis (Table 3) showed that the dressing-type emulsion did not present differences of significance (*p* < 0.05), with values between 6.65 and 6.94 of pH, 0.053 and 0.075% citric acid, and 2.40 and 3.01 °Brix as soluble solids. The samples had a high moisture content (between 64 and 71%). The employed hydrocolloids decreased the fat content of the samples with values of 17.59 and 20.03% while preserving the carbohydrate and protein content.

Lightness (L*), redness (a*), yellowness (b*), chroma (C*), hue angle ($h^{{}^{\circ}}$), and total color difference (ΔE) of the dressing-type emulsions stabilized with hydrocolloids from butternut squash seeds are presented in Table 4. All samples showed different values for L* when the samples with HBSS presented lower values (ES2, ES3, and ES4) than the control sample (ES1). Light scattering of lipid droplets contributes to a milky white or creamy appearance, one of the most desirable properties of many emulsion-based products [46]. The difference in lightness might be due to the varying amounts of light scattering between samples. Light absorption and scattering rely on the refractive index, concentration, size, and dispersion of the droplets, but also on the presence of chromophoric materials [47]. Absorption is primarily responsible for chromatics (redness, greenness, and blueness), while scattering is mainly responsible for the turbidity, lightness, or opacity of an emulsion [47]. It was hypothesized that the different lightness among dressing emulsion samples was most likely due to the differences in particle size and dispersion of droplets, which alter the light segregation. The addition of HBSS to the dressing emulsion was evaluated by the total difference in color (E), which increased with the concentration of HBSS. The results indicated that the HBSS used to prepare dressing-type emulsions could affect the color.

#### 2.2.2. Microstructure Analysis

**Figure 2:**
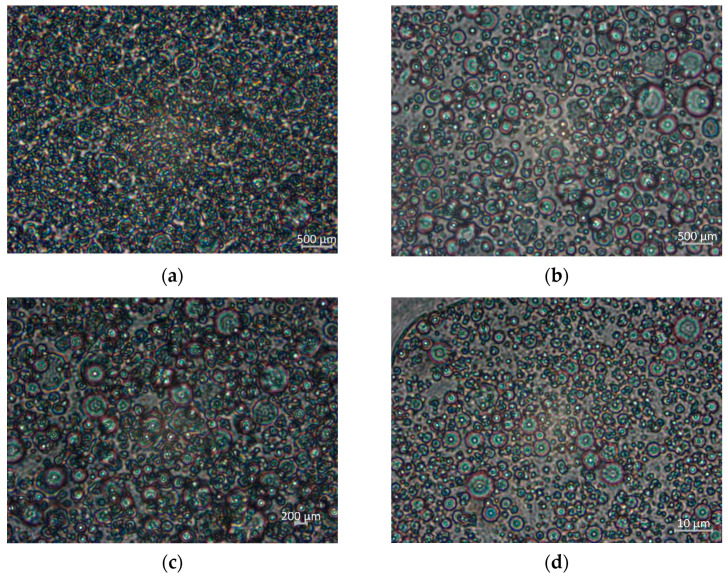
The microscopy properties of the emulsion sauce were evaluated at 100×. (**a**) ES1, (**b**) ES2, (**c**) ES3, and (**d**) ES4.

For the microstructure of dressing-type emulsions (Figure 2), the decrease in oil droplet size is evident with increasing HBSS concentration, presenting a particle size between 3–50 μm. No droplet aggregation was observed, related to the homogenization process that disperses the oils into droplets that spontaneously begin to fuse and promote phase separation in the absence or presence of surfactant in the control samples and those containing hydrocolloids, respectively. The ES1 sample (without HBSS) was stable; however, it presented an agglomeration of droplets; then the smallest particle size and a better droplet distribution were achieved for the hydrocolloid-containing samples. The addition of HBBS improved the stabilization of the samples.

#### 2.2.3. Rheological Properties

**Figure 3:**
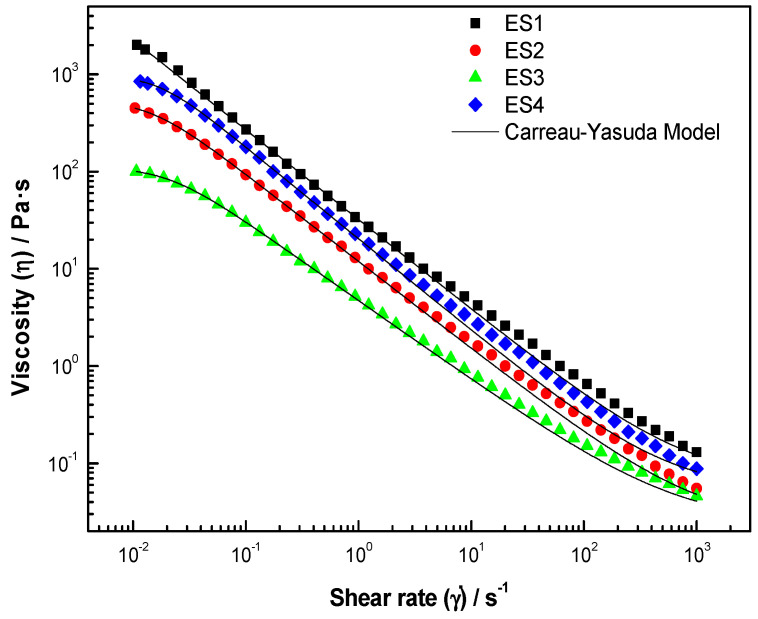
The viscous flow of dressing-type emulsions stabilized with hydrocolloids from butternut squash seeds at pH 10, adjusted to the Carreau–Yasuda model.

Dressing-type emulsions are usually designed to have specific textural characteristics: they should flow from the container but maintain their shape in a salad [8]. The flow curves of the dressing-type emulsions with various ratios of blends of HBSS and XG are shown in Figure 3. The shear stress increased with the increasing shear rate for all samples; thus, all dressing-type emulsions exhibited non-Newtonian behavior. The viscosity of the dressing-type emulsion samples as a function of the shear rate is shown in Figure 3. The viscosity of all samples decreased as the applied share rate increased, representing a similar shear-thinning behavior (pseudoplastic behavior).

The Carreau–Yasuda model (Equation (1)) describes the behavior of the flow of complex systems at high shear rate,

(1) $$\eta =\eta_{\infty }+(\eta_{0}-\eta_{\infty }){\left[1+{\left(\lambda_{c\;}\dot{\gamma }\right)}^{a}\right]}^{\frac{n}{a}}$$

where $\eta_{\infty }$ is the infinite viscosity, $\eta_{0}$ is the viscosity at low shear rate, *a* is the exponent of the power-law, $\lambda_{c}$ is the time constant of Carreau, and *n* is the flux index when n<1 for shear-thinning liquids. The parameters obtained by adjustment to the Carreau model are presented in Table 5. The adjustment adequately reproduces the steady flow behavior of the hydrocolloids, with high correlation coefficients (R^2^ = 0.99).

The adjustment parameters are shown in Table 5. $\eta_{0}$ decreases with the addition of HBSS, and $\eta_{\infty }$ did not show significant differences (*p* > 0.05). λ variated with the HBSS; this difference was observed in samples with 0.5 and 0.75% of HBSS (ES3 and ES4, respectively); *a* values presented different significant (*p* > 0.05) samples with HBSS, indicating the viscous nature of the fluid. In all cases, n was less than one (0.81–0.94), confirming the pseudoplastic nature of the dressing-type emulsion [48], related to distraction and fracture of the droplet or three-dimensional network [49].

**Figure 4:**
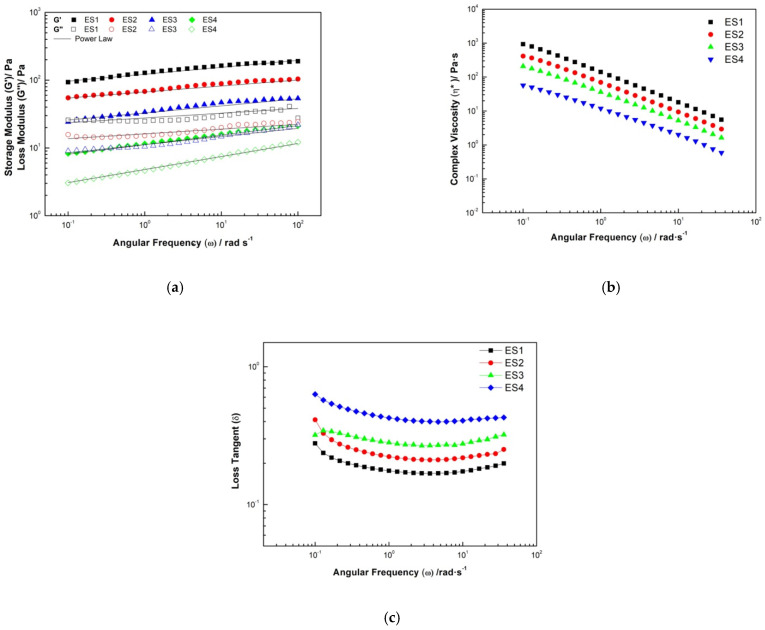
Mechanical spectra obtained in the dynamic oscillatory test of the dressing emulsion stabilized with hydrocolloids from butternut squash seeds at pH 10. (**a**) Loss and storage modulus, (**b**) complex viscosity, and (**c**) loss tangent.

Viscoelastic properties of dressing-type emulsions stabilized with various ratios of blends of HBSS and XG, expressed as storage modulus ($G^{\prime }$), loss modulus ($G^{\prime \prime }$), complex modulus ($G^{*}$), complex viscosity ($\eta^{*}$), and loss tangent (Tan δ) in the linear viscoelastic range, are depicted in Figure 3. The typical evolution of the linear viscoelastic functions with frequency for dressing-type emulsions is also shown. For all samples, $G^{\prime }$ values were higher than $G^{\prime \prime }$ (Figure 4a), indicating a dominant elastic behavior compared to viscous behavior. This indicated their solid-like nature [50]. The result was related to the three-dimensional network formed by the interaction between droplets in the dressing emulsion; this characteristic was found in most Pickering emulsions and other emulsions [51]. Clearly, for all samples, both moduli demonstrated weak frequency dependence, which supported their solid-like performance.

The experimental linearity of the moduli indicated that dressing-type emulsions might be considered as gel-like networks; the $G^{\prime }$ values for all samples increased with frequency due to strong interactions between the droplets that contribute to the elastic modulus, which needed a long time to relax. This might be due to the strong interactions of droplets in the samples. To analyze the effect of the percentage of oil and starch additions on the frequency of $G^{\prime }$ and $G^{\prime \prime }$, the curves were fitted to power-law using Equations (2) and (3) [52]:

(2) $$G^{\prime }=k^{\prime }\omega^{n^{\prime }}$$

(3) $$G^{\prime \prime }=k^{\prime \prime }\omega^{n^{\prime \prime }}$$

where the magnitude of $G^{\prime }$ and $G^{\prime \prime }$ are represented by the values of network stiffness ($k^{\prime }$) and the consistency coefficient ($k^{\prime \prime }$); the exponents $n^{\prime }$ and $n^{\prime \prime }$ are the slopes representing the relationship between modulus and frequency, i.e., when the exponent value is far from zero, it represents a characteristic behavior of a weak gel [53,54].

The power-law parameters ($k^{\prime }$, $k^{\prime \prime }$, $n^{\prime }$, and $n^{\prime \prime }$) present a high correlation coefficient (R^2^ ≥ 0.88), indicating that the power-law model can accurately describe the behavior of the *G*′ and *G*″ modules (Table 6). The magnitudes of $n^{\prime }$ and $n^{\prime \prime }$ did not present a significant difference (*p* > 0.05), corroborating that both modules slightly depend on the frequency studied. In all cases, $n^{\prime }$ and $n^{\prime \prime }$ presented values higher than 1, which were lower than those reported for a Maxwellian fluid ($n^{\prime }$ = 2 and $n^{\prime \prime }$ = 1). Generally, the values of $k^{\prime }$ were far greater compared with the $k^{\prime \prime }$ values, and with HBSS (*p* < 0.05), the quantities of these two parameters decreased significantly, which may be related to the growth of stiffness in systems mainly due to a decrease in repulsive forces and therefore more intramolecular interactions between biopolymer droplets at a higher concentration of HBSS [53,55].

The complex viscosity (η*) decrease with frequency (Figure 4b) suggests the existence of a network that breaks down with increasing frequency, a behavior analogous to the apparent viscosity decreasing with an increasing shear rate in the shearing stress-rate of shear tests and decreasing with the addition of HBSS (*p* < 0.05), exhibiting non-Newtonian behavior. The loss tangent (Tan δ) presented values between 0.17 to 0.42 (Figure 4c) and showed an increase in the percentage of HBSS (*p* < 0.05), which corroborated the behavior of the weak gel (i.e., Tan δ values larger than 0.1), which is typical of dressing and mayonnaise emulsions [56], which means there was no adequate time for network droplets to adapt themselves to the frequency applied during the oscillation period [12,53]. Other researchers reported the same observation (an approximately similar value of Tan δ) when using starch and non-starch hydrocolloids [53,57,58].

## 3. Conclusions

The hydrocolloids of the butternut squash seeds were obtained in acid (pH 3), neutral (pH 7), and alkaline (pH 10) media with extraction yields of 4.68 ± 0.12, 21.17 ± 0.32, and 31.96 ± 0.27%, respectively; in addition, it presented a good protein-carbohydrate ratio, which is an interesting alternative for the development of the microstructure product. Hydrocolloids present rheological properties of gel-type products, with the storage modulus ($G^{\prime }$) always higher than the loss modulus ($G^{\prime \prime }$) in the entire range of angular frequency.

The dressing-type emulsion prepared with HBSS at pH 10 presented physical stability for 15 days. The HBSS did not modify the physicochemical properties (pH, SS, and acidity) but did decrease the fat content and preserve the carbohydrate and protein content. The color parameters of the samples gave evidence that light scattering of lipid droplets contributes to a milky white or creamy appearance; however, the addition of HBSS slightly affected the color and decreased the particle size, with values between 3 and 50 μm, improving their stability. The emulsion exhibited non-Newtonian properties with shear-thinning behavior; the decrease in pseudoplasticity suggests fewer interactions and entanglements in the emulsion system with the addition of HBSS. For all samples, $G^{\prime }$ values were higher than $G^{\prime \prime }$, indicating a dominant elastic behavior more than a viscous behavior, which indicated their solid-like nature, corroborating the loss tangent (Tan δ) values (0.17 to 0.42). The complex viscosity suggests the existence of a network that breaks down with increasing frequency, a behavior analogous to the apparent viscosity decreasing with an increasing shear rate in the shear stress-rate of shear tests and decreasing with the addition HBSS. Dressing-type emulsions are designed to present specific rheological properties that allow them to flow from the container but maintain their shape on a salad.

## 4. Materials and Methods

### 4.1. Materials

Butternut squash was purchased at the local food market in Cartagena (Colombia). Ethanol (99.5% purity) and hexane were obtained from Panreac (Barcelona, Spain). NaOH, acetic acid, and phenolphthalein were purchased from Sigma–Aldrich (St. Louis, MO, USA). Lecithin and xanthan gum were purchased from Tecnas (Medellin, Colombia). Pasteurized and homogenized milk and skim milk powder were purchased from a local Colombian market. All other reagents were of analytical grade.

### 4.2. Hydrocolloid Extraction

Hydrocolloid extraction was carried out following the procedures described by Quintana et al. [26] and López-Barraza et al. [59], with some modifications. The squash seeds were washed with sodium hypochlorite 100 ppm for 5 min and distilled water; after that, the seeds were degreased and ground to maximize the surface area for extraction. A ratio of 1:8 of ground seed mixture: water at pH 3 (acid medium), 7 (neutral medium), and 10 (alkaline medium), was continuously agitated at 80 °C for 4 h. The resulting mixture was separated by centrifugation for 15 min at 4000 rpm, and the supernatant was recollected. For isolating the polymeric hydrocolloids, the extract supernatant was added in a 1:1 ratio of 99.5% ethanol (analytical grade) to precipitate its biopolymer fraction, followed by centrifugation, and freeze-dried at −50 °C and 0.02 Pa for 48 h, employing Labconco Freezone 1.5 L Benchtop Freeze Dry equipment, obtaining the hydrocolloids from butternut squash (*Cucurbita moschata*) seeds (HBSSs). The extraction yield (%) was calculated by the weight parentage of the dried hydrocolloids in the seeds used. Data were measured in triplicate.

### 4.3. Formulation of Dressing Type Emulsion

Dressing-type emulsions were prepared following the procedures described by Quintana et al. [21], with some modifications. Sunflower oils (20%), distilled water (80%), and lecithin (0.5%) as emulsifiers, and blends of xanthan gum (XG) and hydrocolloids from butternut squash seeds (HBSSs) (1%) as stabilizers, were employed. Emulsion premixtures were prepared by thoroughly dispersing the lecithin and desired amount of XG and HBSS in deionized water and stirring at 2500 rpm for 20 min and 30 °C for hydration. Emulsion continuous phases were performed prior to the oil phase of the premixture and homogenization using a homogenization ultra-turrax (IKA T25 basic, Deutschland, Germany) with an S25 N–10ST dispersing tool at 16,800 rpm for 10 min at 30 °C. The samples were stored at 4.0 ± 0.5 °C until analysis.

To evaluate the stability of the emulsions, approximately 10 g of the formulated samples were transferred into universal bottles and tightly sealed with a cap. All samples were placed at 4 °C for 15 days. The separation phase and creaming index were recorded and calculated, respectively [33,34]. Measurements were carried out in triplicate (n = 3).

### 4.4. Physicochemical and Proximal Composition

The pH, soluble solids, titratable acidity (expressed as citric acid), moisture, ash, fat protein, carbohydrate, and fiber content of the hydrocolloids and dressing-type emulsions, were determined after 24 h of processing, following the method described by the Association of Official Analytical Chemistry [60]. The color parameters of lightness ($L^{*}$), red-green color $\left(a^{*}:+:\;\mathrm{red};-:\;\mathrm{green}\right)$, and yellow-blue color ($b^{*}:+:\;\mathrm{yellow};-:\mathrm{blue})$ of the dressing-type emulsions were measured by the colorimeter. Chroma (C*), hue angle ($h^{{}^{\circ}}$), and color variation ($\Delta E^{*}$) were calculated by Equations (4)–(6), respectively:

(4) $$C^{*}=\left\lceil (a^{*2}\right)+{\left(b^{*2})\right\rceil }^{1/2}$$

(5) $$h^{{}^{\circ}}=\tan^{-1}\left(b^{*}/a^{*}\right)$$

(6) $$\Delta E^{*}={\left[{\left(L_{m}^{*}-L_{c}^{*}\right)}^{2}+{\left(a_{m}^{*}-a_{c}^{*}\right)}^{2}+{\left(b_{m}^{*}-b_{c}^{*}\right)}^{2}\right]}^{0.5}$$

where the subscript m represents the sample of emulsions formulated with XG and HBSS, and c represents the control sample (ES1).

### 4.5. Rheological Properties

The rheological characterization of the hydrocolloids and dressing-type emulsions was carried out in a controlled-stress rheometer (Modular Advanced Rheometer System Haake Mars 60, Thermo-Scientific, Karlsruhe, Germany), based on Quintana et al. [21], equipped with a coaxial cylinder (with an inner radius of 11.60 mm, outer radius of 12.54 mm, and cylinder length of 37.6 mm). Each sample was equilibrated 600 s before the rheological test to ensure the same thermal and mechanical history for each sample. All rheological tests were performed three times, and the software HAAKE Rheowin^TM^ was used for data collection and analysis.

#### 4.5.1. Steady Shear Analysis

Viscous flow tests were carried out at a steady state, analyzing the variation of apparent viscosity in a range of shear rates between 0.001 and 1000 s^−1^ for 20 min at 25 °C.

#### 4.5.2. Dynamic Oscillatory Test

The measurements of the viscoelastic properties were performed by small-amplitude oscillatory shear (SAOS). Initially, stress sweeps were carried out at a frequency of 1 Hz, applying an ascending series of stress values from 0.001 to 1000 Pa at 5 °C to determine the linear viscoelasticity interval; after that, frequency sweeps were performed to obtain the mechanical spectrum, using a stress value within the linear viscoelastic range, in a frequency range between 10^−2^ and 10^2^ rad·s^−1^. The data recorded include the storage modulus (*G*′), which provides the elastic component, the loss modulus (*G*′′), which is related to the viscous component of the material, and the loss tangent (Tan *δ*), which is the ratio *G*″/*G*′ and provides the ratio of elastic to viscous response of the material under consideration.

### 4.6. Microstructural Properties

A Primo Star optical microscope (Carl Zeiss Primo Star Microscopy GmbH, Jena, Germany) with a 100× magnification lens was used to observe the internal distribution of the emulsions (ca. 50 μL). A DCMC310 digital camera with Scope Photo software (Version 3.1.615) from Hangzhou Huaxin Digital Technology Co., Ltd., Zhejiang, China, attached to the optical microscope, captured images.

### 4.7. Statistical Analysis

The results were analyzed using the Statgraphics Centurion XVI (Statgraphics, Rockville, MD, USA). An ANOVA (unidirectional) test was applied to determine statistically significant differences (*p* < 0.05) between the samples submitted to the characterizations. The flow curve data were analyzed according to the Carreau–Yasuda model, and frequency sweep data were analyzed by the power-law model. Linear and nonlinear regression procedures were made using the OriginLab v9.1 (Microcal Software Inc., Northampton, MA, USA) software with the Levenberg–Marquardt iteration method to fit the nonlinear curves. The goodness of fit was evaluated by the mean of determination coefficient (*R*), standard error (SE), and adjusted correlation coefficient (Adj. *R*^2^). All tests were performed three times.

## Figures and Tables

**Table 1:** Extraction yields (Ys %) and the proximal composition of hydrocolloids from butternut squash seeds at pH 3, 7, and 10.

Proximal Composition	pH 3	pH 7	pH 10
Ys %	4.68 ± 0.12 ^a^	21.17 ± 0.32 ^b^	31.96 ± 0.27 ^b^
Moisture %	4.42 ± 0.56 ^a^	3.82 ± 0.02 ^a^	5.04 ± 0.92 ^a^
Ash %	16.10 ± 0.85 ^a^	6.80 ± 0.92 ^b^	6.97± 0.18 ^b^
Fat %	0.96 ± 0.05 ^a^	6.43 ± 0.05 ^b^	5.83 ± 0.99 ^b^
Protein %	29.43 ± 0.17 ^a^	36.70 ± 0.38 ^b^	39.30 ± 0.21 ^b^
Carbohydrate%	50.05 ± 1.10 ^a^	52.68 ± 0.34 ^a^	52.09 ± 0.23 ^a^

Data are expressed as mean ± standard deviation. Different letters in the same row express statistically significant differences (*p* < 0.05).

**Table 2:** Formulation and physicochemical properties of the dressing emulsion stabilized with hydrocolloids from butternut squash seeds (HBSSs) at pH 10.

Code Sample	Oil %	Water %	Lecithin %	Xanthan Gum %	HBSS %
ES1	20	78.50	0.50	1.00	0
ES2	20	78.50	0.50	0.75	0.25
ES3	20	78.50	0.50	0.50	0.50
ES4	20	78.50	0.50	0.25	0.75

**Table 3:** Proximal composition of the dressing emulsion stabilized with hydrocolloids from butternut squash seeds (HBSSs) at pH 10.

Sample Code	pH	°Brix	Acidity % Citric Acid	Moisture %	Ash %	Fat %	Carbohydrate %	Proteins %
ES1	6.65 ± 0.01 ^a^	3.01 ± 0.02 ^a^	0.075 ± 0.78 ^a^	64.39 ± 0.99 ^a^	1.12 ± 1.25 ^b^	20.03 ± 2.12 ^a^	13.37 ± 2.11 ^a^	0.39 ± 0.01 ^a^
ES2	6.84 ± 0.03 ^a^	2.89 ± 0.01 ^a^	0.055 ± 1.58 ^a^	68.85 ± 1.99 ^b^	1.02 ± 1.90 ^b^	18.93 ± 2.01 ^a^	9.75 ± 0.02 ^a^	0.37 ± 0.01 ^a^
ES3	6.83 ± 0.02 ^a^	2.67 ± 0.03 ^a^	0.053 ± 0.99 ^a^	70.18 ± 1.23 ^b^	0.59 ± 0.76 ^a^	18.07 ± 1.04 ^a^	10.66 ± 1.02 ^a^	0.39 ± 0.01 ^a^
ES4	6.94 ± 0.04 ^a^	2.40 ± 0.01 ^a^	0.066 ± 1.41 ^a^	71.10 ± 2.45 ^b^	0.92 ± 0.68 ^b^	17.59 ± 3.55 ^a^	9.79 ± 1.77 ^a^	0.40 ± 0.01 ^a^

Data are expressed as mean ± standard deviation. Different letters in the same columns represent statistically significant differences (*p* < 0.05).

**Table 4:** The color parameters of the dressing-type emulsion stabilized with hydrocolloids from butternut squash seeds at pH 10.

Sample Code	L*	a*	b*	C*	$h^{{}^{\circ}}$	ΔE
ES1	85.38 ± 0.97 ^c^	1.25 ± 0.02 ^b^	7.48 ± 0.18 ^a^	28.49 ± 1.36 ^b^	1.40 ± 0.01 ^a^	--
ES2	65.57 ± 1.77 ^a^	2.97 ± 0.43 ^c^	13.10 ± 0.31 ^b^	16.47 ± 0.18 ^a^	1.51 ± 0.01 ^a^	4.35 ± 0.62 ^a^
ES3	79.86 ± 4.91 ^b^	1.04 ± 0.02 ^b^	4.94 ± 0.01 ^a^	12.73 ± 0.01 ^a^	1.36 ± 0.01 ^a^	11.67 ± 2.18 ^b^
ES4	81.20 ± 1.80 ^c^	0.34 ± 0.01 ^a^	5.72 ± 0.04 ^a^	89.30 ± 3.98 ^c^	1.37 ± 0.02 ^a^	182.66 ± 8.07 ^c^

Data are expressed as mean ± standard deviation. Different letters in the same columns express statistically significant differences (*p* < 0.05).

**Table 5:** Parameters of adjustment of Carreau–Yasuda model of the dressing emulsion stabilized with hydrocolloids from butternut squash seeds at pH 10.

Sample Code	$\eta_{0}$	$\eta_{\infty }$	λ	a	*n*	R^2^
ES1	2037.54 ± 9.71 ^d^	0.06 ± 0.00 ^b^	89.14 ± 1.63 ^b^	15.78 ± 0.10 ^b^	0.94 ± 0.04 ^a^	0.99
ES2	548.73 ± 7.84 ^b^	0.02 ± 0.00 ^a^	71.06 ± 0.77 ^b^	1.96 ± 0.08 ^a^	0.89 ± 0.01 ^a^	0.99
ES3	111.99 ± 1.20 ^a^	0.02 ± 0.00 ^a^	48.47 ± 0.82 ^a^	1.88 ± 0.07 ^a^	0.81 ± 0.01 ^a^	0.99
ES4	970.31 ± 9.26 ^c^	0.05 ± 0.00 ^b^	59.73 ± 0.48 ^a^	2.43 ± 0.08 ^a^	0.94 ± 0.01 ^a^	0.99

Data are expressed as mean ± standard deviation. Different letters in the same columns express statistically significant differences (*p* < 0.05).

**Table 6:** Viscoelastic properties of the dressing emulsion stabilized with hydrocolloids from butternut squash seeds at pH 10 evaluated at a constant frequency within the linear viscoelastic region in the dynamic oscillatory test. Storage modulus (*G*′), loss modulus (*G*″), tan δ (*G*′/*G*″), and complex viscosity (η *) at 1 rad·s^−1^.

Sample Code	k′ (Pa)	n′ *	R^2^	k″ (Pa)	n″ *	R^2^	η * (Pa·s)	Tan δ
ES1	135.62 ± 1.12 ^a^	1.11 ^a^	0.97	25.70 ± 0.41 ^a^	1.09 ^a^	0.86	141.01 ± 0.01 ^a^	0.17 ± 0.01 ^a^
ES2	65.78 ± 0.76 ^b^	1.13 ^a^	0.97	16.05 ± 0.30 ^b^	1.10 ^a^	0.86	69.61 ± 0.01 ^a^	0.22 ± 0.01 ^b^
ES3	34.01 ± 0.44 ^c^	1.15 ^a^	0.97	9.92 ± 0.13 ^c^	1.14 ^a^	0.96	36.28 ± 0.01 ^a^	0.28 ± 0.01 ^b^
ES4	10.72 ± 0.26 ^d^	1.21 ^a^	0.96	4.70 ± 0.02 ^d^	1.20 ^a^	0.99	11.89 ± 0.01 ^a^	0.42 ± 0.01 ^c^

Data are expressed as mean ± standard deviation. Different letters in the same columns express statistically significant differences (*p* < 0.05). * Standard deviation was less than 0.01.

## Data Availability

Not applicable.
